# Evaluation of the 5th edition of the TNM classification for gastric cancer: improved prognostic value

**DOI:** 10.1054/bjoc.2000.1548

**Published:** 2001-01-01

**Authors:** E Klein Kranenbarg, J Hermans, J H J M van Krieken, C J H van de Velde

**Affiliations:** Departments of Surgery and Medical Statistics, Medical Statistics, Leiden University Medical Center, PO Box 9600, Leiden, RC, 2300, The Netherlands; Department of Pathology, University Hospital Nijmegen, PO Box 9101, HB, 6500, The Netherlands

**Keywords:** TNM classification, gastric cancer, prognosis

## Abstract

The main change in the 5th edition (1997) of the TNM classification for gastric cancer compared to the 4th edition (1987) is the use of the number of involved nodes instead of the location of positive nodes. As a result stage grouping is also altered. A second change is the requirement for the examination of at least 15 nodes to justify the N0 status. Patients with fewer examined negative nodes are unclassifiable (Nx). Data were retrieved from a randomized trial database comparing D1 and D2 dissection and 633 curatively operated patients were included. According to the criteria of the 5th edition, 39% of the node-positive patients had another N stage compared to the 4th: 21% had a lower and 18% had a higher stage. 5-year survival rates according to the 4th edition N0, N1 and N2 groups were respectively 72%, 34% and 27%. According to the 5th edition these percentages were for the N0, N1, N2, N3 and Nx groups respectively 75%, 38%, 19%, 8% and 65%. The former 1987 N1 and N2 group were significantly split into three new N 1997 groups (*P* = 0.006, respectively *P*< 0.0005). The Cox's regression analysis showed the N 1997 classification to be the most important prognostic variable, with a higher prognostic value than N 1987. In addition, the new TNM stage was also a better prognosticator. The requirement for examining at least 15 nodes, however, could not be fulfilled in 38% of all node-negative patients and we found that a minimum of 5 consecutive negative lymph nodes is a reliable number for staging purposes. We conclude that the 5th edition of the TNM classification provides a better estimation of prognosis, however, examination of at least 15 negative regional lymph nodes is too high a threshold and 5 gives similar prognostic value. © 2001 Cancer Research Campaign http://www.bjcancer.com


*British Journal of Cancer* (2001) **84**(1), 64–71
© 2001 Cancer Research Campaign
doi: 10.1054/ bjoc.2000.1548, available online at http://www.idealibrary.com on IDEAL®

### Evaluation of the 5th edition of the TNM classification for gastric cancer: improved prognostic value

**E Klein Kranenbarg[1], J Hermans[2], JHJM van Krieken[3] and CJH van de Velde[1]**

From the Departments of Surgery[1], and Medical Statistics[2], Leiden University Medical Center, PO Box 9600, 2300 RC Leiden, The Netherlands, and the Department of Pathology, University Hospital Nijmegen, PO Box 9101, 6500 HB, The Netherlands

**Summary** The main change in the 5th edition (1997) of the TNM classification for gastric cancer compared to the 4th edition (1987) is the use of the number of involved nodes instead of the location of positive nodes. As a result stage grouping is also altered. A second change is the requirement for the examination of at least 15 nodes to justify the N0 status. Patients with fewer examined negative nodes are unclassifiable (Nx). Data were retrieved from a randomized trial database comparing D1 and D2 dissection and 633 curatively operated patients were included. According to the criteria of the 5th edition, 39% of the node-positive patients had another N stage compared to the 4th: 21% had a lower and 18% had a higher stage. 5-year survival rates according to the 4th edition N0, N1 and N2 groups were respectively 72%, 34% and 27%. According to the 5th edition these percentages were for the N0, N1, N2, N3 and Nx groups respectively 75%, 38%, 19%, 8% and 65%. The former 1987 N1 and N2 group were significantly split into three new N 1997 groups ($P = 0.006$, respectively $P < 0.0005$). The Cox's regression analysis showed the N 1997 classification to be the most important prognostic variable, with a higher prognostic value than N 1987. In addition, the new TNM stage was also a better prognosticator. The requirement for examining at least 15 nodes, however, could not be fulfilled in 38% of all node-negative patients and we found that a minimum of 5 consecutive negative lymph nodes is a reliable number for staging purposes. We conclude that the 5th edition of the TNM classification provides a better estimation of prognosis, however, examination of at least 15 negative regional lymph nodes is too high a threshold and 5 gives similar prognostic value. © 2001 Cancer Research Campaign http://www.bjcancer.com

**Keywords**: TNM classification; gastric cancer; prognosis

Staging forms the most important prognostic factor in (gastric) cancer and is the basis for treatment decisions. The 4th edition of the TNM classification of malignant tumours by the Union Internationale contre le Cancer (UICC) (Hermanek and Sobin, 1987) and the General Rules for the Gastric Cancer Study in Surgery and Pathology by the Japanese Research Society for Gastric Cancer (JRSGC) (Kajitani, 1981; Aiko and Sasako, 1998; Japanese Gastric Cancer Association, 1998) both are based on the *location* of involved lymph nodes to categorize lymph node involvement. Several investigators have proposed other classifications for lymph node involvement not taking into account the location of the involved nodes, but e.g. the number of involved nodes (Wu et al, 1996). N classifications separately for T1, T2 and T3 (Ichikura et al, 1993) for nodal metastatic rate <50% and ≥50% (Kodera et al, 1997) or for ≤25% and >25% (Yu et al, 1997) have also been proposed.

In 1997, the 5th edition of the TNM classification of malignant tumours was published (Sobin and Wittekind, 1997). For gastric cancer the main difference is the importance of the *number* of involved lymph nodes instead of the *location* of the involved (regional) lymph nodes.

The purpose of the present paper is to compare both TNM classifications for their practical usefulness and prognostic value by using data of the Dutch Gastric Cancer Trial (Bonenkamp et al, 1999).

*Received 22 February 2000*
*Revised 25 September 2000*
*Accepted 25 September 2000*

*Correspondence to:* CJH van de Velde

### PATIENTS AND METHODS

### Patients

A total of 1078 patients with gastric cancer were randomized in the Dutch D1–D2 Gastric Cancer Trial between August 1989 and July 1993 and entered by 78 hospitals with 52 affiliated pathology laboratories. All institutions had obtained medical ethical approval. For eligibility, histologically proven adenocarcinoma of the stomach without clinical evidence of distant metastasis was required. Patients had to be younger than 85 years and biologically suitable for D1 as well as D2 dissection. Patients were excluded if they had previous or co-existing malignancies or previous gastrectomy for benign disease.

The lymph nodes were grouped into 4 levels (N1 to N4) according to the guidelines of the JRSGC (Kajitani, 1981). Patients were randomized to undergo a D1 or D2 dissection. D1 dissection included the lymph nodes directly attached to the stomach, whereas D2 required additional dissection of the lymph nodes belonging to the N2 tier. Details of the trial design are published elsewhere (Bonenkamp et al, 1999).

711 patients were resected with a curative intent with macroscopically completely removed tumour, without peritoneal spread nor liver metastasis nor distant lymph node metastasis, as confirmed by frozen section examination of a para-aortic lymph node biopsy (lymph node station number 16). After pathological examination, 14 of the 'curatively' operated patients appeared to have distant lymph node metastasis: 2 in station 13, 2 in station 14 and 11 patients in station 16. Of the remaining 697 patients, 58 patients were considered to have had a R1 resection (microscopical residual tumour): 20 because of positive cytology of abdominal washing, 35 due to resection-line involvement and 3 patients due to positive cytology as well as positive margins. In 5 of the remaining 639 R0 patients, no primary tumour could be found and in one patient the T-stage could not be determined due to inadequate specimen handling. The present report will therefore be based on 633 patients, including 44 patients who died in the post-operative phase (in-hospital death or within 30 days after surgery), since the postoperative mortality was different between the D1 and D2 group (15 versus 29 patients). Survival time was calculated from the date of surgery until death or last follow-up alive. The range in follow-up time of patients still alive was 6.0 to 10.2 years, with a median follow-up of 7.9 years.

### Classifications

In Table 1, two classifications for lymph node staging are given. The 5th TNM edition (1997) contains three changes with regard to the 4th TNM (1987) classification:

1. The hepatoduodenal lymph nodes (station 12) are now classified as regional lymph nodes instead of distant;
2. Histological examination of a regional lymphadenectomy specimen should include 15 or more lymph nodes to justify a pN0 staging;
3. The number of positive regional lymph nodes determines the N stage (1–6 involved nodes; N1, 7–15 involved nodes: N2 and ≥16 positive nodes: N3).

Due to the fact that not all our patients classified as N0 according to the 1987 edition fulfilled the condition of 15 or more lymph nodes examined, we had to create a Nx category, defined as: according the 1987 classification pN0, but <15 lymph nodes examined. The term *node-negative patient* is used for all patients where no positive nodes were found, irrespective of the number of examined lymph nodes. In consequence, the term *node-positive patient* is used for all patients where one or more positive lymph nodes have been found, irrespective of the number of examined lymph nodes.

The TNM stage grouping has changed as a result of the N changes: all N3 patients are classified as stage IV and T4N1 patients were classified as stage IIIb in 1987, but as stage IV in

**Table 1** The 1987 and 1997 UICC TNM classifications for lymph node staging

| N stage | UICC 1987 4th edition | UICC 1987 5th edition |
|---|---|---|
| N0 | No regional lymph node metastasis | Histological examination of a regional lymphadenectomy specimen will ordinarily include ≥15 lymph nodes |
| N1 | Metastasis in perigastric lymph node(s) ≤ 3 cm of the edge of the primary tumour | 1–6 regional lymph node metastases |
| N2 | Metastasis in perigastric lymph node(s) > 3 cm from the edge of the primary tumour or in lymph nodes along the left gastric, common hepatic, splenic or coeliac arteries | 7–15 regional lymph node metastases |
| N3 | – | ≥16 regional lymph node metastases |
| M1 | Metastasis in hepatoduodenal, retropancreatic, mesenteric or para-aortic nodes | Metastasis in retropancreatic, mesenteric or para-aortic nodes |

UICC: Union Internationale contre le Cancer (Hermanek and Sobin, 1987; Sobin and Wittekind, 1997).

1997 (Table 2). As said before, we had to make additional to the official 1997 TNM definitions, a Nx category and consequently an 'unclassifiable' TNM category for the node-negative patients with <15 lymph nodes examined (TNMx).

### Statistics

The $\chi^2$ test was applied to assess differences in the distribution of patients among groups. The Mann-Whitney test was used for comparison of continuous variables. Univariate survival analysis was carried out by using the Kaplan-Meier method and differences between groups were compared by the log-rank test. The Cox's proportional hazards model with forward stepwise regression was

**Table 2** TNM stage classifications according to the 1987 and 1997 UICC editions

**TNM stage classification 1987**

| | | M0 | | | M1 |
|---|---|---|---|---|---|
| | | N0 | N1 | N2 | |
| M0 | T1 | Ia | Ib | II | IV |
| | T2 | Ib | II | IIIa | IV |
| | T3 | II | IIIa | IIIb | IV |
| | T4 | IIIa | IIIb | IV | IV |
| M1 | | IV | IV | IV | IV |

**TNM stage classification 1997**

| | | M0 | | | | | M1 |
|---|---|---|---|---|---|---|---|
| | | N0 | N1 | N2 | N3 | Nx | |
| M0 | T1 | Ia | Ib | II | IV | x | IV |
| | T2 | Ib | II | IIIa | IV | x | IV |
| | T3 | II | IIIa | IIIb | IV | x | IV |
| | T4 | IIIa | IV | IV | IV | x | IV |
| M1 | | IV | IV | IV | IV | IV | IV |

(shaded) Changes compared to 4th edition (1987); Nx: <15 negative regional nodes examined: x: unclassifiable.

used for multivariate analyses taking into account the following variables: age, gender, tumour location, differentiation grade, T stage, 1987 and 1997 lymph node status, resection type, dissection type and splenectomy/pancreatectomy. Analyses were performed with the Statistical Product and Service Solutions, SPSS 9.0 for Windows, 1998, SPSS Inc., Chicago IL.

### RESULTS

### N stage

In Table 3, the 1987 and 1997 N classifications are summarized and compared. For only 185 (62%) of the 298 node negative patients a minimum of 15 lymph nodes were retrieved. As a result, 113 patients could not be staged, which is 18% of the total 633; they are reported under Nx. From the 213 former N1 patients, 20% migrated: 17% to N2 and 3% to N3. From the former N2 patients, 71% moved: 56% to N1 and 15% to N3. Two patients from the M1 group moved to a regional lymph node status, since station 12 became a regional location: 1 patient moved to N3 and 1 patient to N2. From the 236 new N1 patients, 28% came from N2 and from the N2 patients 51% came from N1. In total, 131 out of 335 of node positive patients (39%) migrated to a lower (21%) or higher (18%) N status.

The prognosis of the different N groups is illustrated in Figure 1. The 5-year survival rates in the 1987 N0, N1 and N2 groups were 72%, 34% and 27%. The difference between N1 and N2 was not significant. The 5-year survival in the N0, N1, N2, and N3 groups of the 1997 N staging was respectively 75%, 38%, 19% and 8%. These curves imply that the new N stages are better discriminators; prognosis between N1 and N2 was significantly different ($P = 0.0006$). Both the N1 1987 as well as N2 1987 group were significantly separated into new N 1997 groups ($P = 0.0063$, respectively $P < 0.0005$). The new N 1997 groups on the contrary are homogeneous with respect to their N 1987 components: they do not significantly differ from each other (Figure 2).

### D1 vs. D2 dissection

Our patient population consists of patients who underwent different dissection types. According to the 1987 classification, a N1 status corresponds with a D1 dissection and a N2 status with a D2 dissection. As a result the N status between the dissection groups was significantly different ($P < 0.0001$), e.g. 32% of D2 patients were N2, compared to only 8% of the D1 patients (Table 4).

**Table 3** Relationship between the 1987 and 1997 lymph node (N) classifications in 633 gastric cancer patients

| | | N 1987 | | | | |
|---|---|---|---|---|---|---|
| | | **Total** | **N0** | **N1** | **N2** | **M1** |
| | Total | 633 | 298 | 213 | 120 | 2 |
| | N0 | 185 | **185** | | | |
| | N1 | 236 | | **169** | 67 | |
| N 1997 | N2 | 73 | | 37 | **35** | 1 |
| | N3 | 26 | | 7 | 18 | 1 |
| | Nx | 113 | 113 | | | |

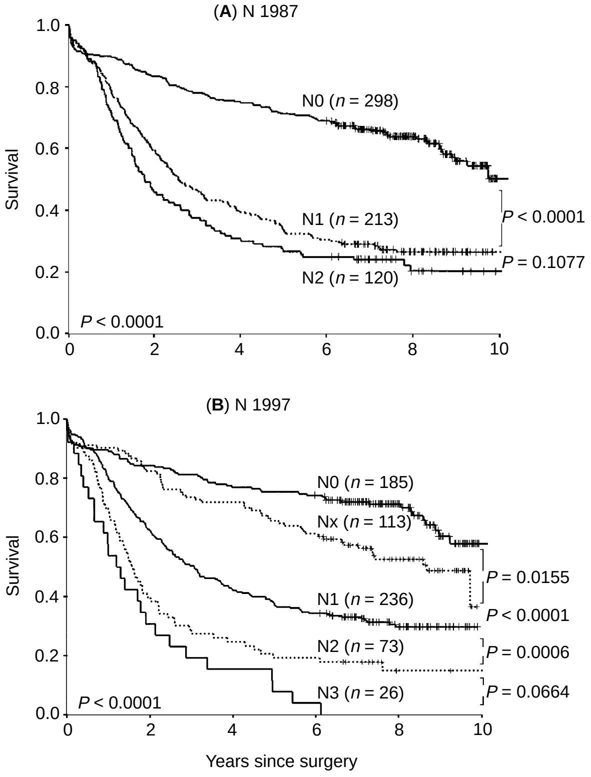

**Figure 1** Survival in 633 patients, according to N 1987 stage (**A**) and N 1997 stage (**B**)

**Table 4** For 633 gastric cancer patients the N status according to the 4th (1987), the 5th official (1997) edition and the 5th modified (1997) edition of the TNM classification

| | **1987** | | | | **1997, official** | | | | **1997, modified** | | | |
|---|---|---|---|---|---|---|---|---|---|---|---|---|
| | **D1** | | **D2** | | **D1** | | **D2** | | **D1** | | **D2** | |
| | ***n*** | **(%)** | ***n*** | **(%)** | ***n*** | **(%)** | ***n*** | **(%)** | ***n*** | **(%)** | ***n*** | **(%)** |
| N0 | 161 | (48) | 137 | (46) | 74 | (22) | 111 | (38) | 147 | (44) | 132 | (45) |
| N1 | 148 | (44) | 65 | (22) | 129 | (38) | 107 | (36) | 129 | (38) | 107 | (36) |
| N2 | 28 | (8) | 92 | (31) | 36 | (11) | 37 | (12) | 36 | (11) | 37 | (12) |
| N3 | – | – | – | – | 11 | (3) | 15 | (5) | 11 | (3) | 15 | (5) |
| Nx | – | – | – | – | 87 | (26) | 26 | (9) | 14 | (4) | 5 | (2) |
| M1 | 0 | (0) | 2 | (0.3) | – | – | – | – | – | – | – | – |
| Total | 337 | (100) | 296 | (100) | 337 | (100) | 296 | (100) | 337 | (100) | 296 | (100) |
| | $P < 0.0001$ | | | | $P < 0.0001$ | | | | $P = 0.28$ | | | |

% represent column percentages.
1997, official classification: ≥ 15 lymph nodes need to be examined to justify a N0 status.
1997, modified classification: ≥ 5 lymph nodes need to be examined to justify a N0 status.

© 2001 Cancer Research Campaign

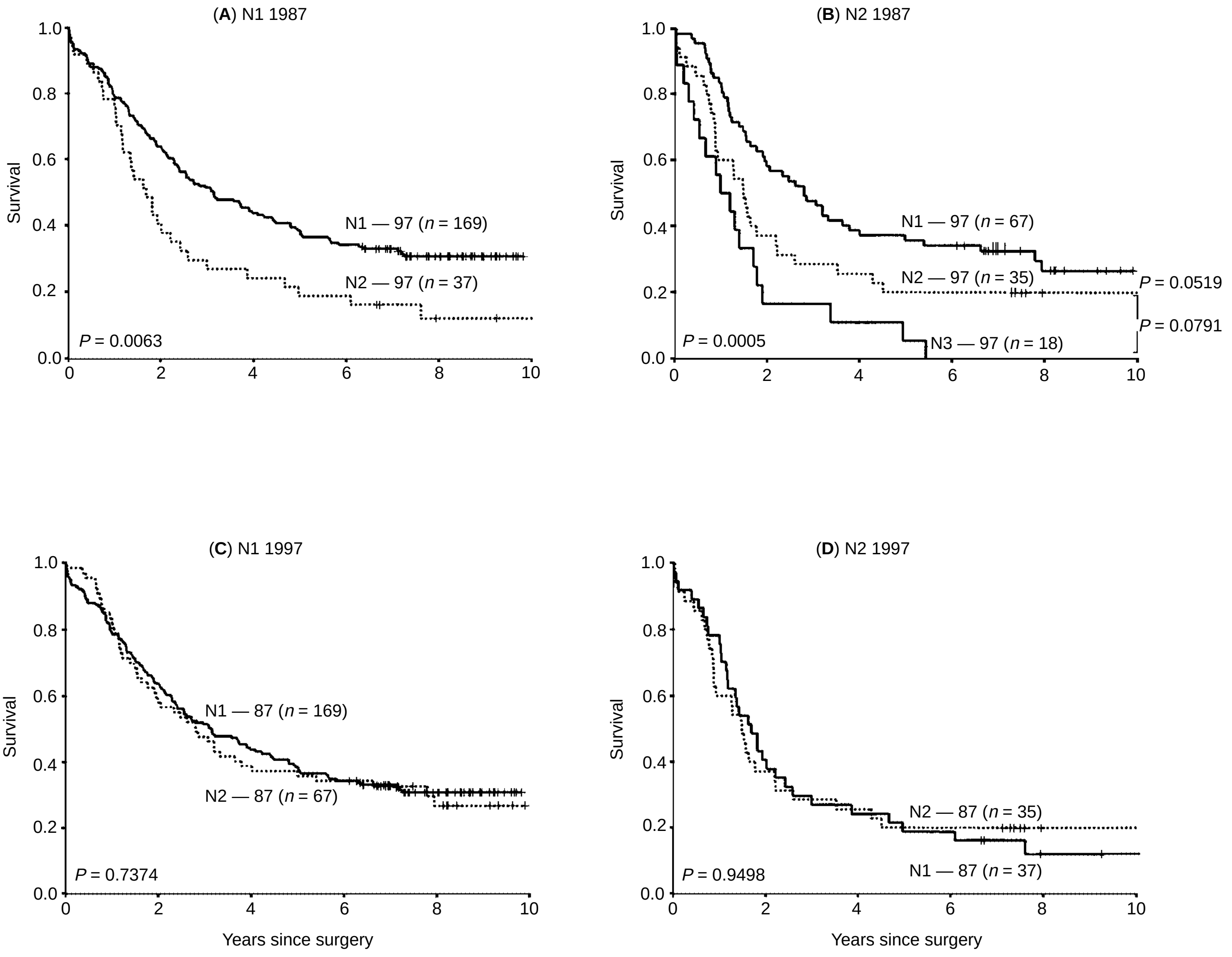

**Figure 2** Survival in node positive patients, according to N 1987 stage (**A** and **B**) and N 1997 stage (**C** and **D**). In figure **A**, the curve for N3–97 ($n$ = 7) is not shown. In figure **D**, the curve for M1–87 ($n$ = 1) is not shown

According to the 1997 classification, N stage was also different between the two dissection groups: 87 out of 161 (54%) D1 node-negative patients were classified as Nx, compared to only 26 (19%) D2 node-negative patients. This is due to the fact that the median number of examined nodes was 15 in the D1 group and 27 in the D2 group ($P < 0.0001$). If the requirement of the number of examined lymph nodes is neglected (N0 + Nx combined), there is no statistical difference in N stage between the D1 and D2 group ($P = 0.58$). The median number of positive lymph nodes on the other hand was 4 in both dissection groups and not significantly different.

### TNM stage

The new N status also resulted in a new TNM stage (Table 5). As a consequence of the Nx status, a TNMx group had to be created. 23% of the non-TNMx patients (120 out of 520) migrated to another stage. From former stage IIIa 50% of patients moved: 25% to stage II, 14% to stage IIIb and 11% to stage IV. From former stage IIIb 69% of patients migrated: 45% to stage IIIa and 24% to stage IV. In the 1987 edition, 10 patients were classified as stage IV, compared to 35 patients in the new edition.

**Table 5** Relationship between the 1987 and 1997 TNM stage distribution in 633 gastric cancer patients

| | | | TNM 1987 | | | | | |
|---|---|---|---|---|---|---|---|---|
| | | **Total** | **Ia** | **Ib** | **II** | **IIIa** | **IIIb** | **IV** |
| | Total | 633 | 143 | 155 | 157 | 123 | 45 | 10 |
| | Ia | 91 | **91** | | | | | |
| | Ib | 112 | | **101** | 11 | | | |
| TNM | II | 151 | | 2 | **118** | 31 | | |
| 1997 | IIIa | 100 | | | 18 | **61** | 20 | 1 |
| | IIIb | 31 | | | | 17 | **14** | |
| | IV | 35 | | | 2 | 13 | 11 | **9** |
| | x | 113 | 52 | 52 | 8 | 1 | | |

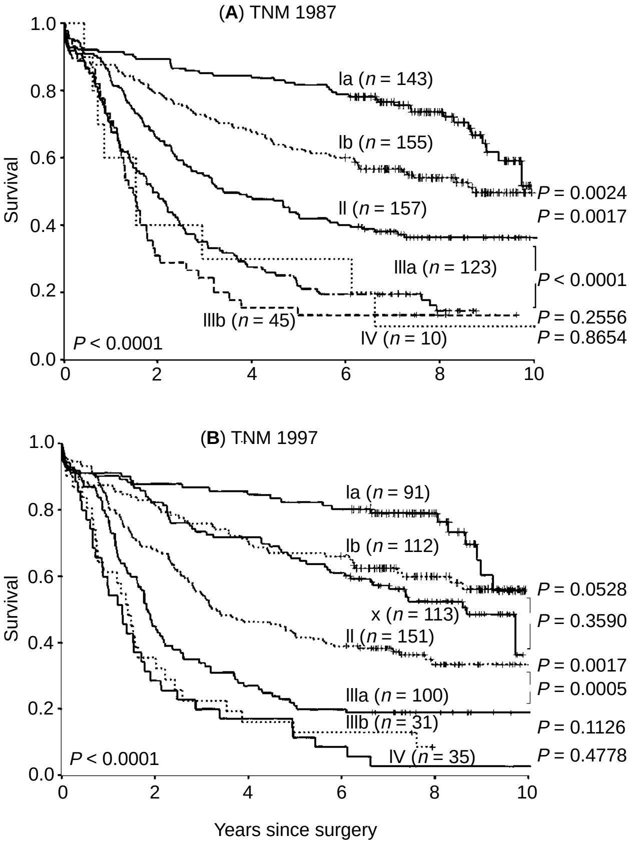

**Figure 3** Survival in 633 patients, according to TNM 1987 stage (**A**) and TNM 1997 stage (**B**)

Survival according to the old and new TNM stage distribution is presented in Figure 3. The survival per stage group was quite similar in both editions. The TNMx group had approximately the same survival as the Ib group. The former stage II, IIIa and IIIb groups are composed of significantly different patients as shown by the new TNM stage distribution ($P$ = 0.05, $P$ = 0.15 and $P$ = 0.02 respectively) (figures not shown). The new stage II, IIIA, IIIb and IV groups are homogeneously composed of former stage II to IV patients (data not shown; not significant).

### Prognostic factors

We evaluated the two N classifications on the one side and on the other side a series of classical prognostic factors: age, gender, T stage, differentiation grade and resection type. The stepwise selection pointed to the N 1997 classification as being more prognostic than the N 1987 classification and moreover N 1997 was selected before all other variables. Of course, as soon as N 1997 was selected, the N 1987 classification did not have much additional prognostic power. But running the model without the N 1997 classification, the most important variable was the N 1987 classification. So N 1987 in itself is highly prognostic, but N 1997 is, according to this Cox's regression analysis, superior (Table 6).

The Nx group had independent prognostic value with RR = 1.69 (95% CI 1.17–2.45), indicating the importance of a minimum requirement in the new classification.

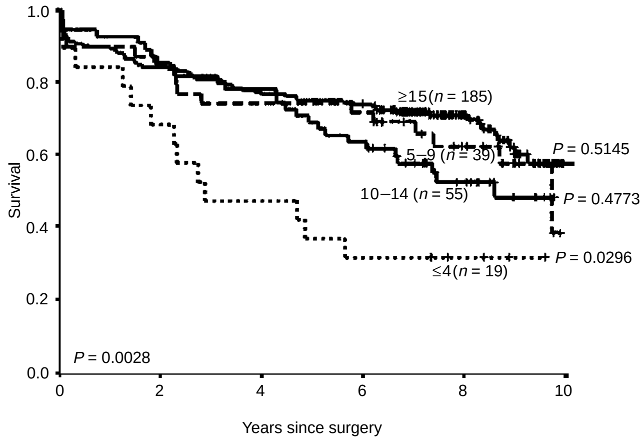

**Figure 4** Survival in 298 node-negative patients separated by number of examined lymph nodes

We also investigated the possibility of a different prognostic value of the N 1997 classification for D1 and D2 separately. For Nx patients, the prognostic value for D1 and D2 was the same (RR = 1.73 respectively RR = 1.87). However, for patients with N2 disease, the relative risk was 6.33 for D1 patients compared to 2.37 for D2 patients. For N3 patients, this difference was also present: RR = 9.95 for D1 patients compared to RR = 4.03 for D2 patients. So it seems that the prognostic value of the N 1997 classification is more important for D1 patients than for D2 patients. In addition, the model selected gender as a prognostic factor in D2

**Table 6** Relative risk and 95% confidence intervals for the fitted multivariate model, in order of stepwise selection

| Variable | *n* | RR | 95% CI | *P* value |
|---|---|---|---|---|
| N 1997 | | | | |
| N0 | 185 | 1 | | |
| Nx | 113 | 1.69 | 1.17–2.45 | 0.0054 |
| N1 | 236 | 2.50 | 1.84–3.39 | <0.0001 |
| N2 | 73 | 3.41 | 2.34–4.96 | <0.0001 |
| N3 | 26 | 4.57 | 2.81–7.43 | <0.0001 |
| Age | 633 | 1.04 | 1.03–1.05 | <0.0001 |
| T stage | | | | |
| T1 | 181 | 1 | | |
| T2 | 308 | 1.55 | 1.14–2.11 | 0.0050 |
| T3 | 132 | 2.36 | 1.66–3.35 | <0.0001 |
| T4 | 12 | 2.04 | 1.00–4.13 | 0.0487 |
| Resection type | | | | |
| Partial | 430 | 1 | | |
| Total | 203 | 1.50 | 1.20–1.88 | 0.0005 |
| Gender | | | | |
| Female | 273 | 1 | | |
| Male | 360 | 1.38 | 1.11–1.71 | 0.0033 |
| Differentiation | | | | |
| Good | 66 | 1 | | |
| Moderate | 208 | 0.97 | 0.65–1.45 | 0.8946 |
| Poor | 340 | 1.36 | 0.93–1.99 | 0.1155 |
| Undifferentiated | 19 | 1.03 | 0.52–2.02 | 0.9373 |

RR: Relative risk, adjusted for all other variables, CI: confidence interval. Dissection type, N 1987 and splenectomy/pancreatectomy were not selected by the model.

 © 2001 Cancer Research Campaign patients (RR = 1.65 for males), although this variable was not selected in D1 patients.

### Cut-off point

In our series, 38% of 298 node-negative patients had to be classified as Nx. A closer look at these patients showed that 92% of these patients had T1 or T2 tumours. To overcome such a high percentage of missing N stages, we have searched for a lower, acceptable minimum number of harvested lymph nodes on the basis of prognosis. In Figure 4, a curve is given for the 298 node-negative patients according to the official 1997 UICC N classification, requiring at least 15 examined lymph nodes in order to justify the node-negative classification. 5-year survival for patients with at least 15 negative nodes was 75%. For patients with 10–14 or 5–9 examined nodes the 5-year survival was still high at 74% (NS). Modifying this rule to the requirement of the consecutive examination of at least 5 negative lymph nodes to justify a N0 classification, led to only 19 unclassifiable patients, moreover with a poor prognosis. The 279 patients who were N0 according to this modified rule, had a prognosis very similar to the 185 official N0 patients. Cox regression analysis with this modified classification showed that the N0 group with ≥15 examined lymph nodes had a similar relative risk than the N0 group with 5–15 examined negative lymph nodes (RR = 1.46, 95% CI 0.98–2.18) was not statistically different. The modified Nx group still had a worse prognosis with a relative risk of 2.48 (95% CI 1.36–4.52) compared to the N0 group with ≥15 examined lymph nodes. Thus, a modification to ≥5 lymph nodes being examined to justify N0 led to only 19 patients out of 633 (3%) with missing N status or 6% of the node-negative patients only.

### DISCUSSION

TNM classifications partly based on the number of positive nodes are already in use in many cancer types. The 1997 TNM classification for gastric cancer has now adopted the number of involved nodes for classifying nodal involvement. Several investigators had already proposed these changes for many years (Kim et al, 1992,1995; Ichikura et al, 1993; Wu et al, 1996). In 1993, Ichikura et al compared the JRSGC, the 4th TNM classification and the number of nodes and concluded that patients with 1–3 positive nodes had as good a prognosis as those without nodal involvement when analysed per T-stage (Ichikura et al, 1993). A multivariate analysis in patients with 4 or more positive nodes showed that the number of positive nodes was the most important prognostic determinant and that N stage (1987) was not significantly prognostic. Authors from Korea also proposed the number of involved nodes (0, 1–3 and 4) (Kim et al, 1995). Others have proposed to use metastatic rate (Kodera et al, 1997; Yu et al, 1997). We also found that the new TNM classification based on the *number* of positive nodes gives better prognostic information than the old TNM classification based on *location* of positive nodes. In 1998, two German groups (Hermanek et al, 1998; Roder et al, 1998) and one Japanese group (Kodera et al, 1998) also concluded that the 1997 TNM classification had led to a better estimation of outcome.

The second major change in the new TNM classification is the requirement to examine at least 15 lymph nodes to classify a patient as N0. In the Dutch gastric cancer trial, from which our data were recruited, meticulous harvesting of lymph nodes was performed (Bunt et al, 1996). However, in 54% of the node-negative patients in the D1 group less than 15 lymph nodes were harvested; even in the D2 group, 19% of node-negative patients did not fulfil the minimum requirement to justify the N0 status. This requirement led to a substantial number of unclassifiable patients, in this report denoted by Nx patients. We have interpreted the rules of ≥15 examined lymph nodes as an adequate number to validate the absence of regional lymph node metastasis. However, it is unclear whether 6 of 6 positive nodes represents Nx or N1 in the 1997 TNM system. We interpreted it as N1, although the true N stage is probably N2 or greater. In our series in fact, in the D1 group we found 67 N1 patients (52%) and 7 N2 (19%) patients with <15 examined nodes. In the D2 group, we found 18 N1 patients (17%) and 0 N2 patients with <15 examined nodes.

Fortunately, we found no relation between dissection (D1 or D2) and number of positive nodes. The German and Japanese studies (Hermanek et al, 1998; Kodera et al, 1998) did not experience such problems: in the first German study (Hermanek et al, 1998) only 2% of patients were unclassifiable and therefore excluded and 9% in the study performed by Roder et al (1998) while it was not mentioned by the authors of the Japanese study (Kodera et al, 1998). In 1999, 3 other Japanese groups showed the superiority of the 1997 classification compared to the Japanese classification, but they also excluded 19%, respectively 9% of the patients who did not fulfil the criterion (Fujii et al, 1999; Ichikura et al, 1999; Kato et al, 1999) In the series of two European groups, however, the new TNM did not improve prognostic stratification and they also excluded patients who did not undergo a D2 dissection (de Manzoni et al, 1999; Mendes-de-Almeida et al, 1999). In summary, 6 out of 8 publications showed that the 1997 classification was superior to the *location* classifications in predicting prognosis of patients. Yet, none of the authors debated the number of 15 nodes. In contrast to our series, all investigators presented results on single institution or academic cohorts, where an extended lymphadenectomy (D2) is standard of care. Our group was composed of patients entered by 78 different, mainly non-academic hospitals, and therefore more representative for daily practice, at least in The Netherlands, although the trial focus was on lymph nodes, so daily practice might even be worse. In the trial, the nodes were removed by a specially trained study coordinator or by one of the 8 supervising surgeons. They divided the perigastric tissue into proper lymph node stations. Afterwards, the tissue was examined further by the pathologist, using the conventional technique, i.e. lamellation and palpation of the specimen.

In a publication on an US gastric adjuvant protocol no statement regarding the lymph nodes was made in 17% of cases, D0 resections were performed in 54% of patients and in 45% of patients less than 11 lymph nodes had been removed. In these patients, 45% of lymph nodes was found to be positive, compared to only 13% when more than 40 nodes were removed, thus the greater the chance of resecting to a level of negative lymphatic (Estes et al, 1998).

The rationale for the examination of at least 15 nodes is based on two German reports (Hermanek, 1991; Wagner et al, 1991). Firstly, Wagner and colleagues (Wagner et al, 1991) determined the number of regional lymph nodes indicated by the JRSGC in 30 non-cancer cadavers. They found 17–44 lymph nodes after a D2 dissection, with an average of 27 nodes and advocated the use of these yields as normal reference values. In the cadaver study, however, the tissue was cleared by dissolving fatty tissue, making lymph nodes of 1 mm visible and a D2 dissection was performed, both making the number for daily routine use extremely high. In a

© 2001 Cancer Research Campaign small number of patients in our study, we have also used the fat clearance technique and found significantly more lymph nodes in the D1 group (mean 25 versus 15, $P < 0.01$) compared to the routinely used techniques (Bunt et al, 1996). Others have shown that comprehensive fat-clearing doubled total lymph node counts, identified smaller lymph nodes, and identified more histologically involved nodes of smaller size and upstaged 29% of specimen (Candela et al, 1990).

Secondly, data from the Erlangen Cancer Center showed that with an increasing number of examined regional lymph nodes a higher incidence of proven node-positive cases and also a higher 5-year survival rate was observed (Hermanek, 1991). These findings were confirmed by another German study (Siewert et al, 1993). They prospectively registered and analysed gastrectomies with extended lymphadenectomies in 19 hospitals, of which 16 were university hospitals and concluded that radical lymphadenectomy (> 26 nodes) improved survival rate compared to < 26 nodes. They concluded that a cut-off point of 15 lymph nodes should be chosen where the prevalence of node-positive patients did not depend on the number of examined lymph nodes anymore. Since this was not a randomized study, it is not clear why > 26 nodes were removed and results were not corrected for potential bias.

Our proposal is to modify the requirement of having at least 15 lymph nodes examined in order to justify a N0 classification into the requirement of at least 5 lymph nodes. At this cut-off point, only 6% of the node-negative patients would be unclassifiable (Nx and TNMx), compared to 38% when using the 15 node threshold. The lack of a survival difference between the modified classification and the original classification supports this cut off point. Determination of a specified minimum number of lymph nodes using conventional techniques was also studied in colorectal patients where at least 12 nodes are required and it was found that at least 7 nodes should be examined (Caplin et al, 1998).

The 1997 TNM classification does not make recommendations on the techniques for the retrieval and examination of the nodes, however fat-clearance (Candela et al, 1990) and lymph node revealing solution (Koren et al, 1998) will increase the yield of the number of examined nodes. Serial sectioning (Isozaki et al, 1997), immunohistochemical staining (Ishida et al, 1997) and more recently reverse transciptase-polymerase chain reaction (RT-PCR) (Mori et al, 1995) will increase the detection of occult metastases.

To comply with the rules of the TNM classification is hardly feasible applying a D1 dissection. However, our recently published long-term survival results of the randomized comparison of D1 versus D2 dissection did not support the routine use of D2: 5-year survival was 45% after D1 and 47% after D2 dissection. Moreover, cumulative relapse risk was not different: 43% after D1 and 37% after D2 dissection (Bonenkamp et al, 1999). Similar results were observed in a randomized MRC trial comparing D1 and D2: no differences could be found concerning overall survival, gastric cancer survival and recurrence-free survival (Cuschieri et al, 1999).

In conclusion, the 5th edition of the TNM classification provides a better estimation of long-term survival, although the cut-off point of at least 15 examined lymph nodes to justify a N0 status caused many unclassifiable patients in our nation-wide trial. We propose the modification to use 5 lymph nodes as a reliable minimum for staging purposes.

### REFERENCES

Aiko T and Sasako M (1998) The new Japanese classification of gastric carcinoma: points to be revised. *Gastric Cancer* **1**: 25–30

Bonenkamp JJ, Hermans J, Sasako M and van de Velde CJH, for the Dutch Gastric Cancer Group (1999) Extended lymph-node dissection for gastric cancer. *N Engl J Med* **340**: 908–914

Bunt AM, Hermans J, van de Velde CJ, Sasako M, Hoefsloot FA, Fleuren G and Bruijn JA (1996) Lymph node retrieval in a randomized trial on western-type versus Japanese-type surgery in gastric cancer. *J Clin Oncol* **14**: 2289–2294

Candela FC, Urmacher C and Brennan MF (1990) Comparison of the conventional method of lymph node staging with a comprehensive fat-clearing method for gastric adenocarcinoma. *Cancer* **66**: 1828–1832

Caplin S, Cerottini JP, Bosman FT, Constanda MT and Givel JC (1998) For patients with Dukes' B (TNM stage II) colorectal carcinoma, examination of six or fewer lymph nodes is related to poor prognosis. *Cancer* **83**: 666–672

Cuschieri A, Weeden S, Fielding J, Bancewicz J, Craven J, Joypaul V, Sydes M, Fayers P, for the Surgical Co-operative group (1999) Patient survival after D1 and D2 resections for gastric cancer: long-term results of the MRC randomized surgical trial. *Br J Cancer* **79**: 1522–1530

de Manzoni G, Verlato G, Guglielmi A, Laterza E, Tomezzoli A, Pelosi G, Di Leo A and Cordiano C (1999) Classification of lymph node metastases from carcinoma of the stomach: comparison of the old (1987) and new (1997) TNM systems. *World J Surg* **23**: 664–669

Estes NC, MacDonald JS, Touijer K, Benedetti J and Jacobson J (1998) Inadequate documentation and resection for gastric cancer in the United States: a preliminary report. *Am Surg* **64**: 680–685

Fujii K, Isozaki H, Okajima K, Nomura E, Niki M, Sako S, Izumi N, Mabuchi H, Nishiguchi K and Tanigawa N (1999) Clinical evaluation of lymph node metastasis in gastric cancer defined by the fifth edition of the TNM classification in comparison with the Japanese system. *Br J Surg* **86**: 685–689

Hermanek P (1991) Onkologische Chirurgie/Pathologisch-anatomische Sicht. *Langenbecks Arch Chir Suppl Kongressbd* 277–281

Hermanek P and Sobin LH (1987) In: Hermanek P, Sobin LH (eds) *UICC TNM Classification of malignant tumours*, Springer-Verlag: New York

Hermanek P, Altendorf-Hofmann A, Mansmann U, Dworak O, Wittekind C and Hohenberger W (1998) Improvements in staging of gastric carcinoma from using the new edition of TNM classification. *Eur J Surg Oncol* **24**: 536–541

Ichikura T, Tomimatsu S, Okusa Y, Uefuji K and Tamakuma S (1993) Comparison of the prognostic significance between the number of metastatic lymph nodes and nodal stage based on their location in patients with gastric cancer. *J Clin Oncol* **11**: 1894–1900

Ichikura T, Tomimatsu S, Uefuji K, Kimura M, Uchida T, Morita D and Mochizuki H (1999) Evaluation of the New American Joint Committee on Cancer/International Union against cancer classification of lymph node metastasis from gastric carcinoma in comparison with the Japanese classification. *Cancer* **86**: 553–558

Ishida K, Katsuyama T, Sugiyama A and Kawasaki S (1997) Immunohistochemical evaluation of lymph node micrometastases from gastric carcinomas. *Cancer* **79**: 1069–1076

Isozaki H, Okajima K and Fujii K (1997) Histological evaluation of lymph node metastasis on serial sectioning in gastric cancer with radical lymphadenectomy. *Hepatogastroenterology* **44**: 1133–1136

Japanese Gastric Cancer Association (1998) Japanese classification of gastric carcinoma – 2nd English edition. *Gastric Cancer* **1**: 10–24

Kajitani T (1981) Japanese Research Society for Gastric Cancer. The general rules for the gastric cancer study in surgery and pathology. *Jpn J Surg* **11**: 127–139

Kato M, Saji S, Kawaguchi Y, Kunieda K, Sugiyama Y, Takagi Y, Hiraoka T, Kumazawa I and Adachi T (1999) A comparison of the prognostic significance between the number of metastatic lymph nodes and nodal stage in gastric carcinoma. *Hepatogastroenterology* **46**: 3281–3286

Kim JP, Yang HK, and Oh ST (1992) Is the new UICC staging system of gastric cancer reasonable? (Comparison of 5-year survival rate of gastric cancer by old and new UICC stage classification). *Surg Oncol* **1**: 209–213

Kim JP, Hur YS and Yang HK (1995) Lymph node metastasis as a significant prognostic factor in early gastric cancer: analysis of 1,136 early gastric cancers. *Am Surg Oncol* **2**: 308–313

Kodera Y, Yamamura Y, Shimizu Y, Torii A, Hirai T, Yasui K, Morimoto T, Kato T and Kito T (1997) Metastatic gastric lymph node rate is a significant prognostic factor for resectable stage IV stomach cancer. *J Am Coll Surg* **185**: 65–69

Kodera Y, Yamamura Y, Shimizu Y, Torii A, Hirai T, Yasui K, Morimoto T, Kato T and Kito T (1998) The number of metastatic lymph nodes: a promising prognostic determinant for gastric carcinoma in the latest edition of the TNM classification. *J Am Coll Surg* **187**: 597–603

© 2001 Cancer Research Campaign

Koren R, Kyzer S, Levin I, Klein B, Halpern M, Rath Wolfson L, Paz A, Melloul MM, Mishali M and Gal R (1998) Lymph node revealing solution: a new method for lymph node sampling: results in gastric adenocarcinoma. *Oncol Rep* **5**: 341–344

Mendes-de-Almeida JC, Limbert M and Mendes-de-Almeida JM (1999) Does the new TNM classification (1997) improve prognostic stratification in gastric cancer submitted to R0 surgery? *Eur J Surg Oncol* **25**: 280–283

Mori M, Mimori K, Inoue H, Barnard GF, Tsuji K, Nanbara S, Ueo H and Akiyoshi T (1995) Detection of cancer micrometastases in lymph nodes by reverse transcriptase-polymerase chain reaction. *Cancer Res* **55**: 3417–3420

Roder JD, Bottcher K, Busch R, Wittekind C, Hermanek P and Siewert JR (1998) Classification of regional lymph node metastasis from gastric carcinoma. German Gastric Cancer Study Group. *Cancer* **82**: 621–631

Siewert JR, Bottcher K, Roder JD, Busch R, Hermanek P and Meyer HJ (1993) Prognostic relevance of systematic lymph node dissection in gastric carcinoma. German Gastric Carcinoma Study Group. *Br J Surg* **80**: 1015–1018

Sobin LH and Wittekind Ch (1997) In: Sobin LH, Wittekind Ch (eds) *UICC TNM classification of malignant tumours* Wiley-Liss: New York

Wagner PK, Ramaswamy A, Ruschoff J, Schmitz Moormann P and Rothmund M (1991) Lymph node counts in the upper abdomen: anatomical basis for lymphadenectomy in gastric cancer. *Br J Surg* **78**: 825–827

Wu CW, Hsieh MC, Lo SS, Tsay SH, Lui WY and P'eng FK (1996) Relation of number of positive lymph nodes to the prognosis of patients with primary gastric adenocarcinoma. *Gut* **38**: 525–527

Yu W, Choi GS, Whang I and Suh IS (1997) Comparison of five systems for staging lymph node metastasis in gastric cancer. *Br J Surg* **84**: 1305–1309

*© 2001 Cancer Research Campaign*
